# Genetic Characterization of Zika Virus Strains: Geographic Expansion of the Asian Lineage

**DOI:** 10.1371/journal.pntd.0001477

**Published:** 2012-02-28

**Authors:** Andrew D. Haddow, Amy J. Schuh, Chadwick Y. Yasuda, Matthew R. Kasper, Vireak Heang, Rekol Huy, Hilda Guzman, Robert B. Tesh, Scott C. Weaver

**Affiliations:** 1 Institute for Human Infections and Immunity, Center for Tropical Diseases, Department of Pathology, University of Texas Medical Branch, Galveston, Texas, United States of America; 2 United States Naval Medical Research Unit, No. 2, Phnom Penh, Cambodia; 3 National Dengue Control Program, Phnom Penh, Cambodia; Colorado State University, United States of America

## Abstract

**Background:**

Zika virus (ZIKV) is a mosquito-borne flavivirus distributed throughout much of Africa and Asia. Infection with the virus may cause acute febrile illness that clinically resembles dengue fever. A recent study indicated the existence of three geographically distinct viral lineages; however this analysis utilized only a single viral gene. Although ZIKV has been known to circulate in both Africa and Asia since at least the 1950s, little is known about the genetic relationships between geographically distinct virus strains. Moreover, the geographic origin of the strains responsible for the epidemic that occurred on Yap Island, Federated States of Micronesia in 2007, and a 2010 pediatric case in Cambodia, has not been determined.

**Methodology/Principal Findings:**

To elucidate the genetic relationships of geographically distinct ZIKV strains and the origin of the strains responsible for the 2007 outbreak on Yap Island and a 2010 Cambodian pediatric case of ZIKV infection, the nucleotide sequences of the open reading frame of five isolates from Cambodia, Malaysia, Nigeria, Uganda, and Senegal collected between 1947 and 2010 were determined. Phylogenetic analyses of these and previously published ZIKV sequences revealed the existence of two main virus lineages (African and Asian) and that the strain responsible for the Yap epidemic and the Cambodian case most likely originated in Southeast Asia. Examination of the nucleotide and amino acid sequence alignments revealed the loss of a potential glycosylation site in some of the virus strains, which may correlate with the passage history of the virus.

**Conclusions/Significance:**

The basal position of the ZIKV strain isolated in Malaysia in 1966 suggests that the recent outbreak in Micronesia was initiated by a strain from Southeast Asia. Because ZIKV infection in humans produces an illness clinically similar to dengue fever and many other tropical infectious diseases, it is likely greatly misdiagnosed and underreported.

## Author Summary

Zika virus (ZIKV) is a mosquito-transmitted flavivirus found in both Africa and Asia. Human infection with the virus may result in a febrile illness similar to dengue fever and many other tropical infections found in these regions. Previously, little was known about the genetic relationships between ZIKV strains collected in Africa and those collected in Asia. In addition, the geographic origins of the strains responsible for the recent outbreak of human disease on Yap Island, Federated States of Micronesia, and a human case of ZIKV infection in Cambodia were unknown. Our results indicate that there are two geographically distinct lineages of ZIKV (African and Asian). The virus has circulated in Southeast Asia for at least the past 50 years, whereupon it was introduced to Yap Island resulting in an epidemic of human disease in 2007, and in 2010 was the cause of a pediatric case of ZIKV infection in Cambodia. This study also highlights the danger of ZIKV introduction into new areas and the potential for future epidemics of human disease.

## Introduction

Zika virus (ZIKV) is a member of the Spondweni serocomplex within the genus *Flavivirus*, family *Flaviviridae* [1]. Other mosquito-borne flaviviruses of public health importance include yellow fever, dengue, St. Louis encephalitis, West Nile and Japanese encephalitis viruses. Although research efforts have focused on many of these viruses, other medically important members of the mosquito-borne flaviviruses, such as ZIKV, have received far less attention.

Zika virus was first isolated from a sentinel rhesus monkey placed in the Zika Forest near Lake Victoria, Uganda in April 1947; a second isolation from the mosquito *Aedes africanus* followed at the same site in January 1948 [2]. Since that time, sporadic isolations have been made from humans and a variety of mosquito species in both Africa and Asia, with studies of human and animal seroprevalence confirming this distribution (Table 1). Zika virus is most likely maintained in a sylvatic cycle involving non-human primates and mosquitoes [3], [4], with cyclic epizootics in monkeys reported in Uganda [5], [6], [7], [8]. In the sylvatic transmission cycle, humans likely serve as incidental hosts. However, in areas without non-human primates, humans probably serve as primary amplification hosts and potentially as reservoir hosts if their viremia is sufficient in duration and magnitude [9]. Although it is thought that enzootic ZIKV is maintained primarily in a monkey/mosquito transmission cycle, antibodies have been detected in numerous other animal species including water buffalo, elephants, goats, hippos, impala, kongoni, lions, sheep, rodents, wildebeest, and zebras [5], [10].

**Table 1:** Probable distribution of Zika virus based on virus isolation and seroprevalence.

Country	Earliest Report*	Seroprevalence† (Humans)	Virus isolation (Human)	Seroprevalence† (Non-human primates)	Virus isolation (Non-human primates)	Virus isolation (Mosquito)	Reference(s)
Borneo	1951	X		*Pongo pygmaeus*			[29], [30]
Burkina Faso	1981					*Aedes aegypti, Ae. furcifer, Ae. jamoti, Ae. opok*	[31], [32]
Cambodia	2010		X				[15]
Cameroon	2010	X					[33]
Central African Republic	1968	X	X			*Ae. africanus, Ae. opok*	[31], [32], [34]
Cote d'Ivoire	1980					*Ae. africanus, Ae. flavicollis, Ae. furcifer, Ae. grahami, Eretmapodites inornatus, Ae. opok, Er. quinquevittatus, Ae. taeniarostris, Ae. tarsalis, Ae. vitattus*	[31], [32], [35]
Egypt	1953	X					[36]
Ethiopia	1967	X					[37]
Gabon	1967	X					[38], [39]
India	1952	X					[40]
Indonesia	1951	X					[13], [41]
Kenya	1967	X					[37], [42], [43]
Malaysia	1951	X				*Ae. aegypti*	[28], [29], [44]
Micronesia, Yap Island‡	2007	X	X				[9], [17]
Nigeria	1968	X	X				[45], [46], [47], [48], [49]
Pakistan	1980	X					[10]
Philippines	1953						[50]
Senegal	1968	X	X		*Cercopithecus aethiops, Erythrocebus patas*	*Ae. aegypti, Ae. dalzieli, Ae. fowleri, Ae. furcifer, Ae. luteocephalus, Ae. metallicus, Ae. minutus, Ae. neoafricanus, Ae. tarsalis, Ae. vitattus, Anopheles gambiae, Mansonia uniformis*	[14], [31], [32], [51]
Sierra Leone	1972	X					[52]
Somalia	1967	X					[37], [43]
Tanzania	1948	X					[53]
Thailand	1954	X					[44]
Uganda	1947	X		*Cercopithecus aethiops, Cercopithecus ascanius schmidti, Cercopithecus mona denti, Cercopithecus albigena johnstoni, Colobus abyssinicus*		*Ae. africanus*	[2], [3], [5], [6], [7], [8], [11], [43], [53], [54], [55], [56], [57]
USA	2009	X					[14]
Vietnam	1954	X					[44]

*Earliest report, indicates either the first virus isolation or the first report of seroprevalence.

**†:** Seroprevalence was either determined by one or more of the following methods: Haemagglution inhibition, neutralization, complement-fixation, IgG and/or IgM ELISA. Of note, it is possible due to antigenic cross-reactivity among flaviviruses that seropostive individuals may have been previously exposed to one or more flaviviruses and not to Zika virus.

**‡:** Viral RNA sequenced from four patients (Lanciotti et al. 2008).

Human case reports of clinically diagnosed ZIKV infections include self-limiting acute febrile illnesses with fever, headache, myalgia and rash, similar to that caused by many other arboviruses found throughout the tropics [9], [11], [12], [13], [14], [15]. This clinical picture could easily be mistaken for dengue (DEN) or chikungunya (CHIK) fevers, two common arboviral infections which both produce similar clinical presentations. The latter two infections are much more commonly diagnosed in tropical Africa and Asia than ZIKV. Clinical DENV and CHIKV infections are familiar to local clinicians and most diagnostic laboratories can detect them. In contrast, few physicians are aware of ZIKV and few laboratories test for clinical infection. Consequently, most ZIKV infections are probably missed or incorrectly diagnosed, as suggested by the high prevalence of ZIKV antibodies found in serosurveys of human populations in Africa and Asia (Table 1). A recent epidemic on Yap Island, Federated States of Micronesia, and a pediatric case of ZIKV infection in Cambodia demonstrate that ZIKV is also capable of causing human disease and may be expanding its geographic distribution [9], [15].

Zika virus, has a positive-sense, single-stranded RNA genome approximately 11 kilobases in length [16]. The genome contains 5′ and 3′ untranslated regions flanking a single open reading frame (ORF) that encodes a polyprotein that is cleaved into three structural proteins: the capsid (C), premembrane/membrane (prM), and envelope (E), and seven non-structural proteins (NS1, NS2A, NS2B, NS3, NS4A, 2K, NS4B, and NS5) [16]. A previous genetic study using nucleotide sequences derived from the NS5 gene indicated three ZIKV lineages: East African (one strain examined), West African (three strains examined), and Asian (one strain examined) [17].

Although ZIKV circulates widely in sub-Saharan Africa and Southeast Asia, little is known of the genetic relationships among isolates from these two geographic regions, which may have different vector/host transmission cycles. Furthermore, the geographic origin of the strain responsible for the epidemic on Yap Island epidemic and of the recent Cambodian case of ZIKV infection is unknown. To answer these questions, we determined the nucleotide sequences of the ORF of five ZIKV strains collected between 1947 and 2010 in Cambodia, Malaysia, Nigeria, Uganda, and Senegal and constructed phylogenetic trees to assess their relationships.

## Methods

### Virus strains, RNA preparation, genomic amplification and sequencing

The five strains sequenced in this study were obtained from the World Reference Center for Emerging Viruses and Arboviruses (WRCEVA) at the University of Texas Medical Branch (Table 2). The viruses were passaged in cell culture and harvested following the observation of diffuse cytopathic effect. Viral RNA was extracted from cell culture supernatants using the QIAamp Viral RNA Kit (Qiagen, Valencia, CA, USA). The ORF of the five viruses were amplified using the Titan One Tube PT-PCR System (ROCHE, Mannheim, Germany) and primers designed against conserved sequences to produce overlapping genome segments using African and Asian ZIKV strains: MR 766 (Prototype, Uganda, 1947, GenBank accession number AY632535) and EC Yap (Yap Island, Micronesia, 2007, GenBank accession number EU545988). Purified DNA was then sequenced using the PCR primers and additional internal sequencing primers. The Applied Biosystems BigDye Terminator version 3.1 Cycle Sequencing Kit (Foster City, CA, USA) and the Applied Biosystems 3500 genetic analyzer were used to sequence the amplicons.

**Table 2:** Viruses used in this study.

Isolate	Species/Source	Origin	Year of collection	Passage history	Reference (GenBank accession no.)
MR 766	Sentinel rhesus	Uganda	1947	SM 3	[15] (AY632535)
MR 766	Sentinel rhesus	Uganda	1947	SM 146, C6/36#1	HQ234498‡
MR 766	Sentinel rhesus	Uganda	1947	Unknown	[21] (DQ859059)
P6-740	*Aedes aegypti*	Malaysia	1966	SM 6, Vero 1, BHK 1, C6/36#1	HQ234499‡
IbH 30656	Human blood	Nigeria	1968	SM 21, Vero 1	HQ234500‡
ArD 41519	*Aedes africanus*	Senegal	1984	AP61#1, C6/36#2	HQ234501‡
EC Yap*	Human blood	Micronesia	2007	N/A	[16] (EU545988)
FSS13025	Human blood	Cambodia	2010	Vero 1	JN860885‡
SM-6 V-1†	Unknown	Unknown	Unknown	Unknown	[21] (DQ859064)

AP61 = *Aedes pseudoscutellaris* cells, BHK = baby hamster kidney epithelial cells, C6/36 = *Aedes albopictus* cells, SM = suckling mouse, Vero = African green monkey kidney cells.

*The sequence of this isolate was determined by epidemic consensus (EC) from the viral RNA of four patients (Lanciotti et al. 2008).

**†:** SM-6 V-1 is a strain of Spondweni virus, all other viruses listed within the table are Zika virus strains.

**‡:** Sequenced in this study.

### Genetic and phylogenetic analyses

Nucleotide sequences derived from the five ZIKV strains were assembled and aligned with three other sequences of ZIKV and one sequence of Spondweni virus that were retrieved from GenBank using the Vector NTI Suite (Invitrogen, USA) (Table 2). The two GenBank sequences of the MR766 strain exhibited considerable nucleotide and amino acid variation; therefore we resequenced this strain (Table 2). Phylogenies were generated using neighbor-joining (NJ), maximum-likelihood (ML) and maximum-parsimony (MP) methods using the default settings implemented in the PHYLIP package [18]. The Spondweni virus strain SM-6 V-1 was used as the outgroup for all phylogenies, as Spondweni virus is the most closely related flavivirus (antigenically and genetically) to ZIKV [1], [19], [20], [21], [22]. Robustness of the phylogenies was evaluated by resampling with 1,000 bootstrap replicates and horizontal branches were scaled according to the number of nucleotide substitutions per site.

## Results

### Phlyogenetic analyses

**Figure 1:**
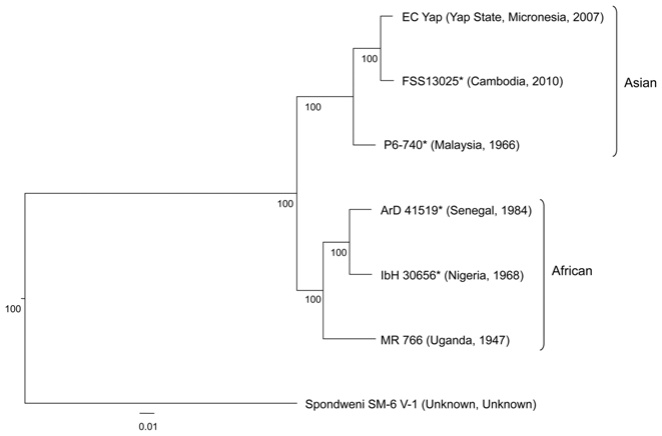
Zika virus nucleotide and amino acid alignments. Neighbor-joining phylogeny generated from open reading frame nucleotide sequences of Zika virus strains. The tree was rooted with Spondweni virus (GenBank accession number DQ859064). The scale at the bottom of the tree represents genetic distance in nucleotide substitutions per site. Numbers at the nodes represent percent bootstrap support values based on 1,000 replicates. Isolates are represented according to strain name, country of origin, and year of isolation. The lineage of each virus is indicated to the right of the tree. *Strains sequenced in this study.

All three methods of phylogenetic inference (NJ, ML and MP) identified two major lineages (African and Asian) (Figure 1). The most recent common ancestor of MR 766 (Uganda, 1947) diverged first, followed by the divergence of the most recent common ancestor of the ArD 41519 (Senegal, 1984) and IbH 30656 (Nigeria, 1968) strains, the P6-740 strain (Malaysia, 1966), and lastly the EC Yap (Micronesia, 2007) and FS13025 (Cambodia, 2010) strains.

### Nucleotide and amino acid sequence variation among African and Asian strains

Based on nucleotide and amino acid sequence composition, the African strains were the most divergent from the Asian strains, and strains from the same geographic regions were the least divergent (Africa and Asia) (Table 3). There were several deduced amino acid differences among the strains, which in turn correlated to geographic area of virus collection.

**Table 3:** Pairwise comparisons of African and Asian Zika virus strains.*

Sequence	MR 766†	IbH 30656	ArD 41519	P6-740	FSS13025	EC Yap
MR 766†		**2.2**	**1.7**	**3.1**	**3.7**	**3.7**
IbH 30656	7.0		**1.2**	**3.1**	**3.6**	**3.9**
ArD 41519	7.0	3.2		**2.7**	**3.1**	**3.4**
P6-740	10.2	10.3	10.1		**1.2**	**1.5**
FSS13025	11.6	11.7	11.6	4.3		**0.8**
EC Yap	11.4	11.7	11.5	4.3	1.8	

*Lightface type = Percent nucleotide divergence; Boldface type = Percent amino acid divergence.

**†:** GenBank accession no. AY632535.

### Genetic variation among three MR 766 sequences

The two MR 766 strains that had been previously sequenced exhibited extensive genetic variation (6.3% nucleotide, 1.8% amino acid divergence) (Table 2). To investigate this discrepancy (Table 2), we sequenced an additional strain of MR 766 from the WRCEVA collection. The MR 766 sequence with accession number AY632535 [16], was ultimately chosen for use in our analyses due to its low passage history, nucleotide and amino acid similarity to the high passage MR 766 strain that we sequenced (0.4% nucleotide and 0.6% amino acid divergence), and its position closest to the root of the MR 766 lineage in a tree including all three sequences (not shown).

### Glycosylation site variation between African and Asian strains

**Figure 2:**
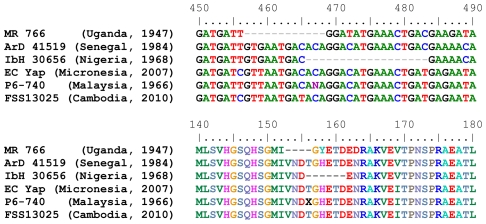
Zika virus phylogeny. Nucleotide (top) and amino acid (bottom) sequences of the envelope protein/gene of Zika virus strains showing the deletions in the potential glycosylation sites of the MR 766 (Uganda, 1947) and the IbH 30656 (Nigeria, 1968) strains. Deletions are indicated by dashes. The “N” at position 467 of the P6-740 strain (Malaysia, 1966) represents an equally weighted double population of the nucleotides “C” and “T”. This translates to an “X” at position 165 of the amino acid alignment.

Deletions in a potential glycosylation site of several strains were observed following their alignment (Figure 2). A 4-codon deletion was observed beginning at amino acid position 153 of the E protein of the MR766 strain (GenBank accession number AY632535), a 6-codon deletion at position 156 of another MR 766 strain (GenBank accession number DQ859059), and a 6-codon deletion at position 156 of the IbH 30656 strain sequenced in this study. The MR 766 strain (Passage 147) sequenced here did not exhibit any deletions in the predicted amino acid sequence and provided evidence of passage-associated changes in potential glycosylation site(s).

## Discussion

### Origin and expansion of Zika virus

Prior to this study, only two ZIKV strains had been genetically characterized [16], [17], [22]. Our phylogentic analyses with the inclusion of five newly sequenced strains revealed the existence of two major ZIKV lineages. Analyses showed the basal divergence of the African and Asian lineages. Furthermore, results of the present study show that the FSS13025 (Cambodia, 2010), P6-740 (Malaysia, 1966) and EC Yap (Micronesia, 2007) strains belong to the Asian lineage, suggesting a recent common ancestor.

The clinical similarity of ZIKV infection to classical DEN fever and CHIK fever may be one reason why this disease has rarely been reported in Asia. During World War II, for example, DEN fever was a major medical problem among Allied and Japanese troops in Southeast Asia and the South Pacific [23]. At that time, there were no specific laboratory tests that could differentiate between the three diseases. In fact, CHIKV and ZIKV still had not been isolated. Consequently, cases of acute febrile illness were likely diagnosed clinically as DEN or possibly as scrub typhus or malaria, diseases that were known and frequently diagnosed among the many foreign military and civilian personnel present in the region at that time [23]. Carey has reviewed the historical confusion in differentiating DEN fever from CHIK fever [24]; but ZIKV infections have probably been misdiagnosed or not reported for some of the same reasons. Given the low level of nucleotide divergence among the ZIKV isolates here (≤11.7%), conserved regions could be utilized for the development of diagnostic assays that will not only aid in detecting new ZIKV infections but to also differentiate them from other arbovirus infections.

Our results strengthen previous epidemiologic evidence that the EC Yap strain originated in Southeast Asia [9], [17]. This conclusion is further substantiated by the geographic proximity of Yap Island to known areas of ZIKV transmission (Indonesia and Malaysia). It has been reported that wind-blown mosquitoes can travel distances of several hundred kilometers over the open ocean [25], [26]. However, due to the great distances involved, it seems likely that the virus was introduced as a result of travel or trade activities whereby either a viremic person, enzootic host species, and/or an infected and subsequently infective mosquito (adult or immature) was transported to the island as suggested by Duffey *et al.* [9]. This hypothesis is further supported by the fact that no monkeys were present on Yap Island during the 2007 epidemic [9].

The phylogenetic results indicate that the Cambodian strain diverged from the Malaysian strain in the recent past. Therefore, the most recent common ancestor of the Cambodian strain has been circulating in Southeast Asia since at least the mid-1900's. These data indicate that Cambodian strain was either recently introduced or that it has been circulating in the region and has remained undetected until 2010. Seroprevalence surveys might help to determine when ZIKV was introduced into Cambodia.

### Glycosylation sites

Several of the ZIKV strains we analyzed exhibited the deletion of a potential N-linked glycosylation site that has been previously described in some ZIKV and West Nile virus strains [16], [17]. It has been hypothesized that extensive mouse brain or cell culture passage could lead the deletion of the potential glycosylation site [27]. Therefore, it is important to note that several of the strains in our analyses had previously undergone mouse brain passages (MR 766, IbH 30656, and P6-740) (Table 2). Of these strains, two different sequences of the MR 766 strain(s) AY632535 and DQ859059 [16], [22], and the IbH 30656 strain sequenced in this study had a deletion in the potential N-linked glycoslyation site. The high passage MR 766 strain that we sequenced, did not exhibit this deletion. These results provide strong evidence that passage history has influenced glycoslyation sites in the MR766 strain. Since all of the MR766 strains have undergone passage in mouse brains it is impossible to determine if the deletion was present in the original strain, as is also the case for the IbH 30656 strain. Further sequencing of geographically distinct, low passage strains that have not undergone mouse brain passage is needed to ultimately resolve whether this glycoslyation site polymorphism occurs in circulating strains or if it only reflects passage history.

### Limitations

We had access to only a small number of ZIKV strains. However, these strains were broadly distributed over time and space, and the phylogenetic analyses were robust. Several of the ZIKV strains had been passaged intracranially in mice, and included two highly passaged strains, IbH 30656 (Nigeria, 1968, passage history: suckling mouse 21, Vero 1) and MR 766 (Uganda, 1947, passage history: suckling mouse 146, C6/36 # 1). It is likely that nucleotide/amino acid changes may have resulted from the passage history of the IbH 30656 strain, which may have slightly influenced the corresponding, terminal branch lengths in our tree but not its overall topology.

### Summary

This investigation indicates that the Yap Island epidemic, which occurred in the Federated States of Micronesia in 2007, most likely resulted from the introduction of a Southeast Asian ZIKV strain(s) pointing to an expansion of the Asian ZIKV lineage. Although ZIKV has one of the earliest and best-documented widespread geographic distributions among arboviruses, many unanswered questions remain concerning its evolution, ecology and epidemiology. In Asia, evidence suggests that the primary mosquito vectors are *Ae. aegypti* and/or *Ae. albopictus* [13], [28], though several ecologically or geographically distinct mosquito vectors may be responsible for the transmission and/or maintenance of ZIKV throughout Asia. As such, further studies are needed to determine the primary and secondary mosquito vectors responsible for ZIKV transmission throughout the Asian region. In addition, human seroprevalence studies throughout Asia may provide insight into the expansion of the Asian lineage and clues as to why certain geographical regions maybe more suitable for virus maintenance and transmission than others. Additional work is needed to better understand the clinical presentation, tropism and pathogenesis of ZIKV infection in humans. Finally, continued ZIKV isolations in currently affected regions coupled with active surveillance in presently naïve areas will allow researchers to follow the possible geographic expansion of the virus and predict the potential emergence of ZIKV into uncharted territories.

### Disclosure Statement

The views expressed in this article are those of the authors and do not reflect the official policy or position of the Kingdom of Cambodia, U.S. Department of Defense, or the Department of the Navy.
